# Characterization of the Yellow Gene Family in *Nilaparvata lugens* and Functional Analysis of *NlYellow-b*

**DOI:** 10.3390/insects17080766

**Published:** 2026-07-25

**Authors:** Xiaohong Zheng, Xinkai Liang, Yiqian Li, Haitao Hu, Shurui Zhang, Ruixue Jia, Yufan Liu, Xiaoli Li, Yi Zhang, Kedong Xu, Xinxin Shangguan

**Affiliations:** 1 Key Laboratory of Plant Genetics and Molecular Breeding, Zhoukou Normal University, Zhoukou 466001, China; xhzheng@126.com; 2 Henan Key Laboratory of Crop Molecular Breeding and Bioreactor, Zhoukou 466001, China; 3 College of Life Science and Agronomy, Zhoukou Normal University, Zhoukou 466001, China; 19139047328@163.com (X.L.); linqier365591988@163.com (Y.L.); 19351751255@163.com (H.H.); 13462082017@163.com (S.Z.); 15637702681@163.com (R.J.); 19838019805@163.com (Y.L.); 4 Henan Plant Gene and Molecular Breeding Engineering Research Center, Zhoukou 466001, China; xiaoli890107@163.com; 5 Henan Crop Molecular Design Breeding and Cultivation Engineering Technology Research Center, Zhoukou 466001, China; yizhang0401@sina.com

**Keywords:** *yellow* gene, *Nilaparvata lugens*, expression analyses, functional analysis

## Abstract

Members of the *yellow* gene family encode conserved Yellow proteins with diverse roles in insect pigmentation, cuticle formation, and reproduction, making them promising targets for RNA interference (RNAi)-mediated pest control strategies. However, little is known about their phylogeny and functions in the brown planthopper (*Nilaparvata lugens*), a devastating rice pest that serves as a vector for viral rice diseases. Here, we identified and characterized 11 *yellow* genes in *N. lugens*, which showed distinct structures and expression profiles. Phylogenetic analysis revealed that *N. lugens* has retained orthologs of *yellow-y*, *-b*, *-d*, *-e*, *-g*, *-g2*, *-h*, and *-x* but lacks those of *yellow-c* and *-f*, suggesting lineage-specific gene loss. Nine of the eleven *NlYellow* genes were localized to chromosome 8, with several positioned in close genomic proximity. The clustering of these loci suggests that tandem duplication events contributed to the expansion of the *NlYellow* gene family. *NlYellow-b* exhibited the highest transcript levels among all identified *NlYellow* genes, particularly in the female ovipositor, and functional characterization via RNAi revealed that it is essential for molting, wing development, and female fertility. Knockdown of *NlYellow-b* in fourth-instar nymphs caused lethal molting defects and wing malformation and significantly reduced offspring production. These findings highlight the dual roles of *NlYellow-b* in development and reproduction, advance our understanding of *yellow* gene function in insects and highlight *NlYellow-b* as a candidate for RNAi-based management of *N. lugens*.

## Simple Summary

We identified 11 *yellow* genes in the *N. lugens* genome and characterized their spatiotemporal expression patterns. Functional analysis via RNAi revealed that *NlYellow-b* plays critical roles in molting, wing development, and female fertility. These findings provide novel insights into the multiple functions of *yellow-b* in *N. lugens* and contribute to a deeper understanding of its biological significance. Given its essential functions in development and reproduction, *NlYellow-b* is a potential molecular target for the development of more effective and sustainable control strategies against *N. lugens*.

## 1. Introduction

Insect Yellow proteins are characterized by a conserved ~300-amino-acid MRJP domain, and major royal jelly proteins (MRJPs) evolved from ancestral Yellow proteins [1]. They were first characterized in *Drosophila melanogaster*, where loss of function produces a yellow-brown cuticle phenotype, highlighting their role in pigmentation [2]. Similarly, larval head cuticle and anal plates of the silkworm *Bombyx mori* are white in the wild type but reddish brown in *Bmyellow-e* mutants [3]. Yellow proteins also regulate pigmentation in insect orders other than Diptera and Lepidoptera. In *Schistocerca gregaria*, RNA interference (RNAi)-mediated knockdown of a yellow protein suppressed the appearance of yellow individuals and yellow staining in the exuviae [4]. In *Henosepilachna vigintioctopunctata*, different *yellow* genes have opposing effects on coloration: *HvY-b* and *HvY-c* inhibited and promoted the coloration of dark markings, respectively, whereas *HvY-e* and *HvY-h* suppressed pigmentation outside the black patches on the body cuticles of adults and larvae [5]. In *Tribolium castaneum*, the *yellow* gene *TcY-e* appears to influence adult body pigmentation, as RNAi knockdown of *TcY-e* caused adult beetles to be slightly darker than the controls. *TcY-e* knockdown also caused lethal desiccation that could be rescued by 100% humidity, suggesting that *TcY-e* has an essential role in cuticle integrity and water retention [6] and that *yellow* genes function in processes other than pigmentation.

*Yellow* genes also modulate insect courtship behavior and reproductive fitness. In *D. melanogaster*, *yellow* (*y*) acts downstream of the *fruitless* gene, a key regulator of the sex-determination pathway that profoundly influences male courtship behavior [7]. Likewise, *yellow* acts as an inhibitory factor in the neural circuitry that governs male courtship in *Bicyclus anynana*, and its knockout increased male courtship behavior [8]. By contrast, *yellow* governs the pigmentation of the male-specific bristle structure—the sex comb—on *D. melanogaster* legs. Loss of *yellow*-mediated pigmentation reduced melanization and altered bristle morphology, impairing the male’s ability to grasp the female genitalia during pre-copulatory behavior. This structural deficit reduced mating efficiency and thus compromised reproductive fitness [9]. Collectively, *yellow* genes are mainly involved in insect pigmentation, cuticle formation, and reproductive processes, including courtship behavior and fecundity, making them potential targets for the development of novel insect-control strategies. 

The brown planthopper, *Nilaparvata lugens* Stål, is a destructive monophagous rice pest that causes substantial yield losses throughout Asia [10,11]. As a phloem feeder, it directly damages plants by extracting sap and also serves as a vector for devastating viral diseases [12,13]. Although chemical control is currently the most common method for *N. lugens* management, resistance has been reported to the majority of commercial pesticides [14]. A potential alternative strategy is plant-mediated RNAi, in which insect-targeting double-stranded RNA (dsRNA) is expressed in crop plants. Compared with the application of conventional protein toxins, this approach offers greater specificity and a more precise mode of action [15], but its success depends largely on the selection of suitable target genes. Given their roles in insect courtship and reproduction, the *yellow* genes represent one such target; however, the *yellow* gene family has not yet been identified genome-wide and functionally studied in *N. lugens*, particularly the key members relevant to pest control.

In this study, we identified and characterized the 11 *NlYellow* genes in the *N. lugens* genome. Among all identified *NlYellow* genes, *NlYellow-b* exhibited not only the highest overall transcript abundance but also pronounced enrichment in the female ovipositor. Subsequent RT-qPCR analysis further confirmed its female-biased and ovipositor-specific expression pattern. Because previous studies have demonstrated that *yellow* family genes often contribute to cuticle formation and reproductive processes, *NlYellow-b* was selected as a representative candidate for functional investigation. Functional characterization of *NlYellow-b* by dsRNA-mediated gene silencing revealed its roles in molting, wing development, and female fertility, making it a potential molecular target for brown planthopper control.

## 2. Materials and Methods

### 2.1. Insect Rearing and Growth Conditions

Brown planthoppers, *N. lugens* (Stål), were initially obtained from the Center for Excellence in Molecular Plant Sciences in Shanghai, China. They were reared on 30-day-old seedlings of the susceptible rice cultivar Taichung Native 1 under controlled environmental conditions (26 ± 2 °C, 16 h light/8 h dark photoperiod) and transferred to new rice seedlings every 14 days to maintain optimal nutritional conditions.

### 2.2. De Novo Identification of NlYellow Genes in N. lugens

The current version of the *N. lugens* genome (NCBI Taxonomy ID: 108931) contains 18,160 protein-coding gene models, annotated on the basis of homology-based and de novo gene predictions. The hidden Markov model (HMM) matrix file for the yellow protein family (PF03022) was downloaded. The HMM profile was then searched against the brown planthopper proteome data with HMMER 3.0 with the default E-value cut-off of 0.001. Each putative yellow protein sequence was checked against the SMART (https://smart.embl.de/, accessed on 22 July 2026), NCBI CDD (https://www.ncbi.nlm.nih.gov/cdd/, accessed on 22 July 2026), and Pfam databases (https://www.ebi.ac.uk/interpro/search/sequence/, accessed on 22 July 2026) to confirm the presence of the MRJP domain. Bioperl v1.7.8 [16] was used to calculate ther physicochemical properties of the NlYellow proteins, including the theoretical isoelectric point, instability index, aliphatic index, and grand average of hydropathicity. 

### 2.3. Phylogenetic Analysis

The yellow proteins from *N. lugens*, *Aedes aegypti*, *B. mori*, *D. melanogaster*, and *T. castaneum* were aligned using MUSCLE [17], and the resulting alignment was used to construct a maximum-likelihood phylogenetic tree with 1000 bootstrap replicates in MEGA 10 [18]. All sequences used for tree construction are provided in Table S1.

### 2.4. Conserved Motifs and Gene Structures

Conserved motifs were identified in the NlYellow proteins using MEME tools (http://meme-suite.org/), with a maximum of 10 conserved domains per gene, a minimum motif width of 15 amino acids, and a maximum motif width of 35 amino acids. The structures of the *NlYellow* genes were predicted using GSDS 2.0 (http://gsds.gao-lab.org/) [19]. TBtools-II v2.486 was used to visualize the conserved motifs, genome annotations, and motif information [20].

### 2.5. Chromosomal Locations

The locations of the *NlYellow* genes on chromosomes of the *N. lugens* reference genome were visualized using TBtools-II v2.486 and plotted onto chromosome location images using MapChart 2.32 [21]. MCScanX v1.0.0 [22] was used to detect syntenic blocks within the *N. lugens* genome, which were then visualized using Circos v 0.69-9 [23].

### 2.6. Gene Expression Analyses

RNA-seq raw data from different developmental stages (eggs, first through fifth instars, and adults; SRP310488) and tissues (fat body, ovary, ovipositor, salivary glands, gut, head, and integument; SRP375431) of *N. lugens* were obtained from NCBI [24]. Expression levels of the *NlYellow* genes were calculated as reads per kilobase of exon model per million mapped reads (RPKM). For heatmap visualization, RPKM values were transformed using log2(RPKM + 1) and normalized by row-wise Z-score transformation. Hierarchical clustering was performed using the Euclidean distance metric with the complete linkage method. Heat maps were generated using TBtools-II v2.486.

All primers used for RT–qPCR were designed using Primer3 v2.6.1 [25] (Table S2). Total RNA was isolated from samples of each tissue using RNAiso Plus reagent (Takara, Dalian, China, Cat no. 9108). First-strand cDNA was obtained by reverse transcription using the PrimeScript RT Reagent Kit with gDNA Eraser (Takara, Dalian, China, Cat no. RR047A) and amplified by qPCR on a Bio-Rad CFX-96 Real-Time PCR system (Bio-Rad Laboratories, Hercules, CA, USA) using the iTaq Universal SYBR Green Supermix Kit (Bio-Rad, CA, USA, Cat no. 1725121). PCR was performed under the following conditions: denaturation for 3 min at 95 °C, followed by 40 cycles at 95 °C for 10 s, and 60 °C for 30 s. A partial fragment of the *N. lugens actin* gene was amplified with qNlActin-F and qNlActin-R primers to serve as an endogenous control for normalization (Table S2). Relative gene expression was calculated using the 2^−ΔΔCt^ method [26].

### 2.7. dsRNA Synthesis and RNAi

*NlYellow-b* and the *green fluorescent protein* (*GFP*) gene from *Aequorea victoria* were targeted for knockdown by RNAi. To minimize the possibility of off-target silencing, the dsRNA fragment was designed from a gene-specific region of *NlYellow-b* and subjected to sequence comparison against other *NlYellow* family members prior to synthesis. DsRNA-specific primers containing T7 promoter sequences were designed, and all primer sequences are listed in Table S2. The correct sequences of *NlYellow-b* and *GFP* were cloned into the pTOPO-Blunt Simple Vector plasmid, which were isolated using the M5 Hiper Multi-color EndoFree Plasmid Mini Kit (Mei5bio, Beijing, China, MF077-01). These plasmids served as templates for PCR amplification using dsRNA-specific primers. The resulting PCR products were used for dsRNA synthesis performed with the MEGA script T7 High Yield Transcription Kit (Ambion, Austin, TX, USA). The quality and concentration of *dsNlYellow-b* and *dsGFP* were assessed using 1% agarose gel electrophoresis and a NanoDrop 2000 spectrophotometer (Thermo Fisher Scientific, Waltham, MA, USA), respectively. The final dsRNA concentrations were adjusted to 5 μg/μL with diethyl pyrocarbonate-treated water.

For the RNAi experiments, fourth-instar nymphs were injected with *dsNlYellow-b* or *dsGFP* (as a control) at the junction between the prothorax and mesothorax using a PV850 microinjector (World Precision Instruments, Sarasota, FL, USA). After injection, the nymphs were transferred to fresh TN1 rice seedlings. To evaluate the efficiency of RNAi, total RNA was extracted from five insects randomly collected at 24, 48, and 96 h after injection and used for RT–qPCR as described above. 

### 2.8. Insect Survival Assays

Fourth-instar nymphs were micro-injected with *dsNlYellow-b* or ds*GFP* as described above, and the number of surviving insects was recorded at the same time each day for nine days. There were five independent replicates for each injection treatment, and each replicate included at least 10 nymphs. The phenotypic characteristics of the injected insects were observed using an optical microscope (Nikon SMZ25, Nikon Corporation, Tokyo, Japan). 

### 2.9. Insect Fecundity Assays

To evaluate the effects of *NlYellow-b* knockdown on reproductive capacity, fifth-instar nymphs injected with *dsNlYellow-b* or ds*GFP* were selected. After emergence, the following cross-mating experiments were performed: *dsNlYellow-b*-injected females × *dsGFP*-injected males and *dsGFP*-injected males × *dsGFP*-injected females (control). For each treatment, nine mating pairs were established, and each pair was maintained individually on fresh TN1 rice seedlings under controlled conditions. Five days after mating, adults were collected and preserved at −80 °C for verification of gene silencing. Hatched nymphs were counted 10 days after the removal of the mating pairs. 

### 2.10. Data Analysis

Statistical analyses were performed using SPSS 18.0 software (IBM, New York, NY, USA). Student’s *t*-test or one-way analysis of variance (ANOVA) was used to compare gene expression data, insect mortality rates, and fecundity rates. Data are presented as the mean ± standard error of the mean (SEM) or the mean.

## 3. Results

### 3.1. Identification and Characterization of NlYellow Genes

A total of 11 genes encoding yellow proteins were identified in the *N. lugens* genome. Their gene ID numbers, genomic lengths, cDNA lengths, protein lengths, molecular weights, isoelectric points, and other properties are provided in Table 1. The NlYellow proteins varied from 289 (NlYellow-d1) to 743 (NlYellow-g1) amino acids in length and from 32.85 kDa to 83.60 kDa in molecular weight, with theoretical isoelectric points between 5.03 and 9.08.

### 3.2. Phylogenetic Analysis of NlYellow Proteins

**Figure 1:**
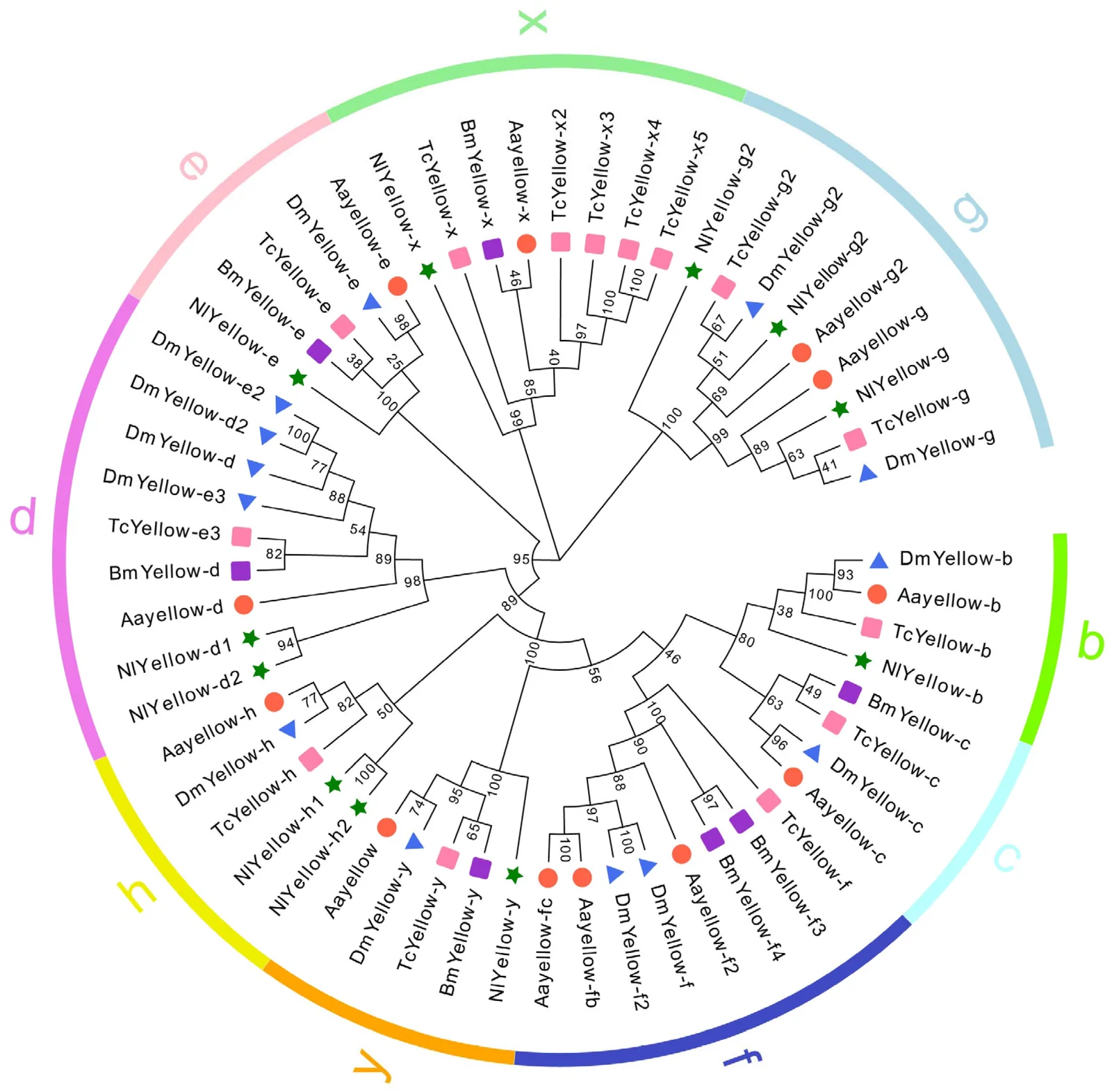
Maximum-likelihood phylogenetic tree of yellow proteins from *Nilaparvata lugens* (Nl), *Bombyx mori* (Bm), *Drosophila melanogaster* (Dm), *Aedes aegypti* (Ae), and *Tribolium castaneum* (Tc). Different colors and point shapes denote species origin: green stars (*N. lugens*), purple squares (*B. mori*), blue triangles (*D. melanogaster*), red circles (*A. aegypti*), and pink squares (*T. castaneum*). Numbers at the nodes are support values from 1000 bootstrap replicates. Major clades (g, x, e, d, h, y, f, c, and b) are indicated in different colors in the outer circle.

We constructed a maximum-likelihood phylogenetic tree of yellow proteins from *N. lugens* (11 proteins), *Aedes aegypti* (12), *B. mori* (8), *D. melanogaster* (13), and *T. castaneum* (14) (Figure 1); all protein sequences used for tree construction are provided in Table S1. Yellow proteins from groups b, c, d, e, f, g, h, x, and y each formed distinct clades with high bootstrap support, indicating their strong evolutionary conservation across these insect species. The 11 NlYellow proteins were named according to their close phylogenetic relationships to facilitate the identification of their orthologs in other species. Proteins previously annotated as both yellow-d and yellow-e3 in *T. castaneum* and *D. melanogaster* fell into a single clade with good support, which should be termed yellow-d [27]. Most *yellow* genes appeared to have undergone additional duplication events in at least one evolutionary lineage. While gene duplications were evident in several clades (e.g., *NlYellow-d1/d2*, *NlYellow-g1/g2/g3*, and *NlYellow-h1/h2*), *N. lugens* lacked orthologs of yellow-f and yellow-c proteins. 

### 3.3. Conserved Protein Motifs and Exon–Intron Organization of NlYellow Genes

**Figure 2:**
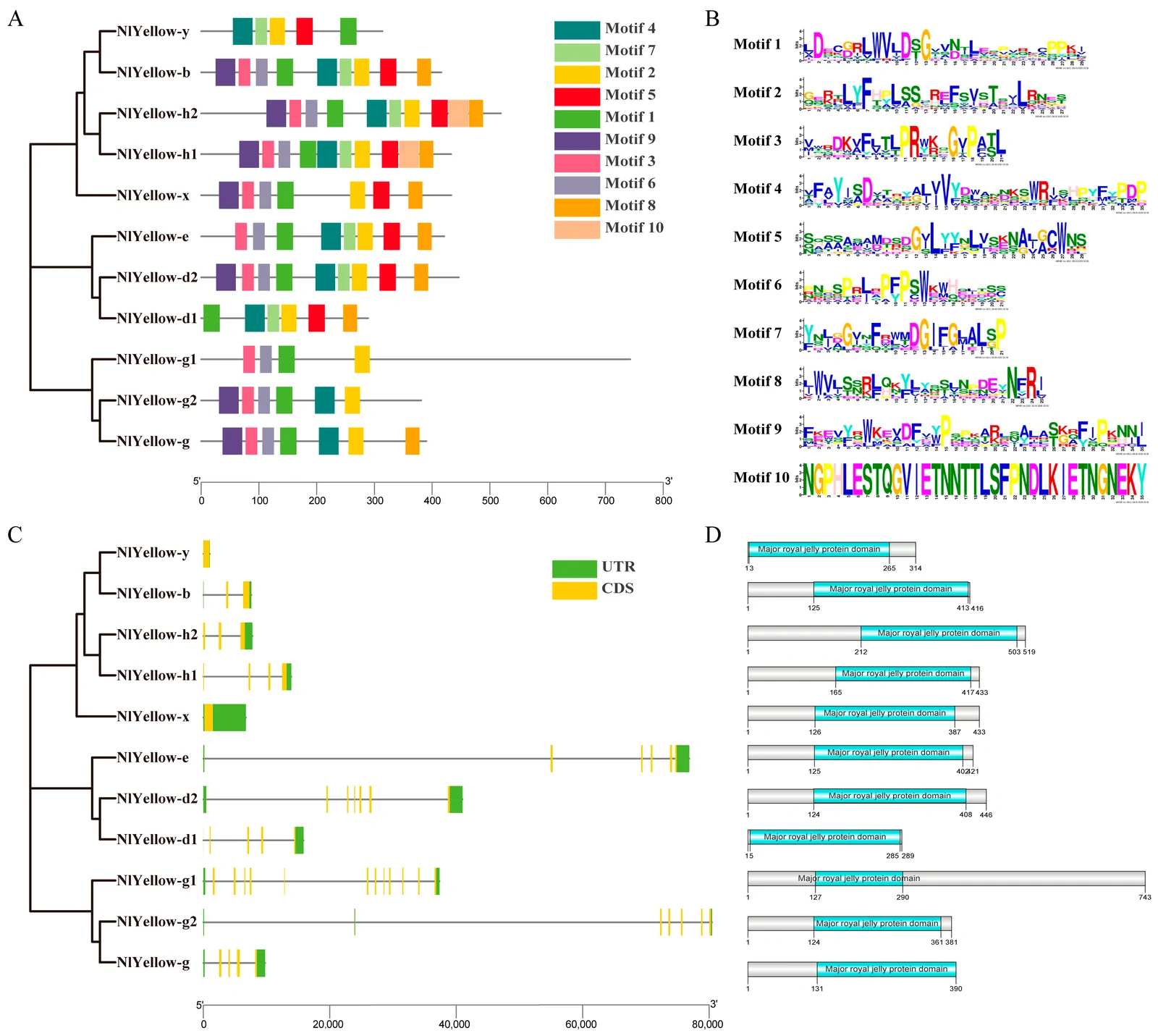
Conserved NlYellow protein motifs and exon–intron structures of *NlYellow* genes. (**A**) Phylogenetic tree and predicted motif composition of NlYellow proteins. (**B**) Web logos depicting the sequences of the 10 conserved motifs shown in (**A**). The height of the stack reflects positional conservation, and the heights of the individual residues indicate their relative frequencies. (**C**) Exon–intron organization of *NlYellow* genes. Yellow bars are exons, green bars are untranslated regions, and black lines are introns. (**D**) Locations of major royal jelly protein domains (blue) in the predicted NlYellow proteins, with junction amino acid residues numbered.

Conserved motifs serve as molecular signatures, facilitating the functional characterization and phylogenetic classification of gene families [28]. We identified 1–10 conserved motifs per protein using the MEME server (Figure 2B, Table S3). Motifs 1 and 2, which were identified in all 11 NlYellow proteins, correspond to the MRJP domain. Their universal presence suggests that they are characteristic motifs of the yellow gene family in *N. lugens*. NlYellow-h1 and NlYellow-h2 contained all ten identified motifs, whereas NlYellow-g1 contained only four motifs (Figure 2A).

We examined the exon–intron boundaries of the *NlYellow* genes to gain insight into their structural organization. Owing to their evolutionary conservation [29] and the principle that structure dictates function [30], these boundaries are important in driving gene family diversification. The exon numbers of the *NlYellow* genes ranged from 1 (*NlYellow-y* and *NlYellow-x*) to 12 (*NlYellow-g1*) (Figure 2C). Analysis of their amino acid sequences confirmed that all NlYellow proteins contain the conserved MRJP domain (~300 amino acids) (Figure 2D), characteristic of insect yellow proteins [31].

### 3.4. Chromosome Locations and Collinearity of the NlYellow Genes

**Figure 3:**
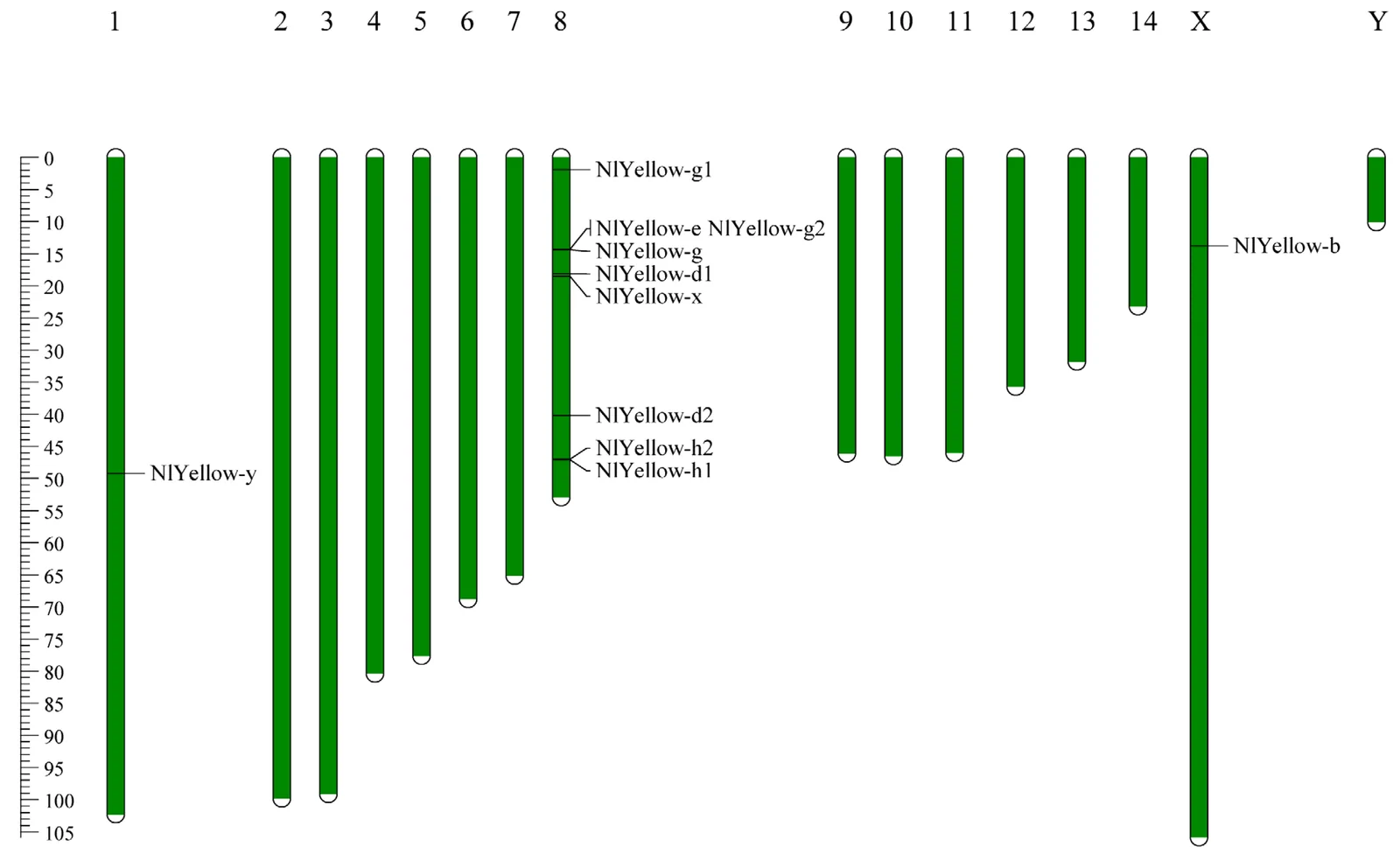
Chromosomal distribution of *NlYellow* genes on the sixteen *N. lugens* chromosomes. The chromosomal position of each *yellow* gene was mapped according to the *N. lugens* genome. The scale bar on the left represents the length (Mb) of the planthopper chromosomes. Chromosomes numbers are labeled at the top of each chromosome.

We next mapped the chromosomal locations of the *NlYellow* genes (Figure 3) and found that they were present on only three of the sixteen *N. lugens* chromosomes. Strikingly, nine of the eleven *NlYellows* were located on chromosome 8, and the remaining two were found on chromosomes 1 (*NlYellow-y*) and X (*NlYellow-b*). 

**Figure 4:**
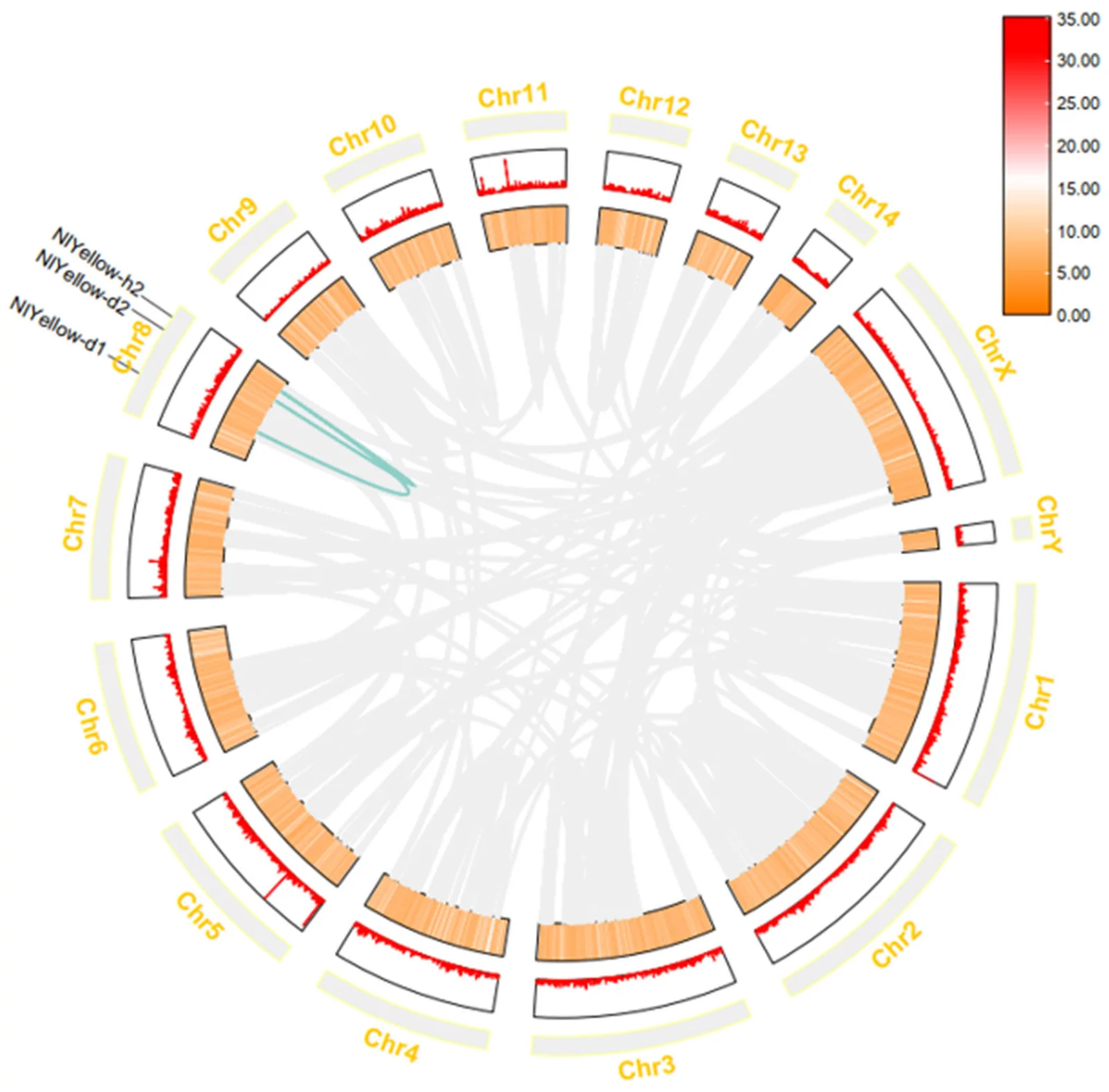
Syntenic relationships among *NlYellow* genes. The scale around the circle is in megabases. Gray lines denote collinear modules within the *N. lugens* genome, and cyan lines specifically highlight syntenic relationships between two pairs of *NlYellow* gene paralogs.

To further clarify the evolutionary history and genomic origins of the *NlYellow* genes, we performed a comprehensive synteny analysis, which identified two significant pairs of duplicated genes arising from tandem duplications: (i) *NlYellow-h2*/*NlYellow-d2* and (ii) *NlYellow-d1*/*NlYellow-d2* (Figure 4). The observed chromosomal bias and collinearity in *NlYellow* gene distribution suggest that these genomic arrangements may reflect important functional constraints or evolutionary adaptations that have been maintained through selection pressures.

### 3.5. Expression Profiles of the NlYellow Genes

**Figure 5:**
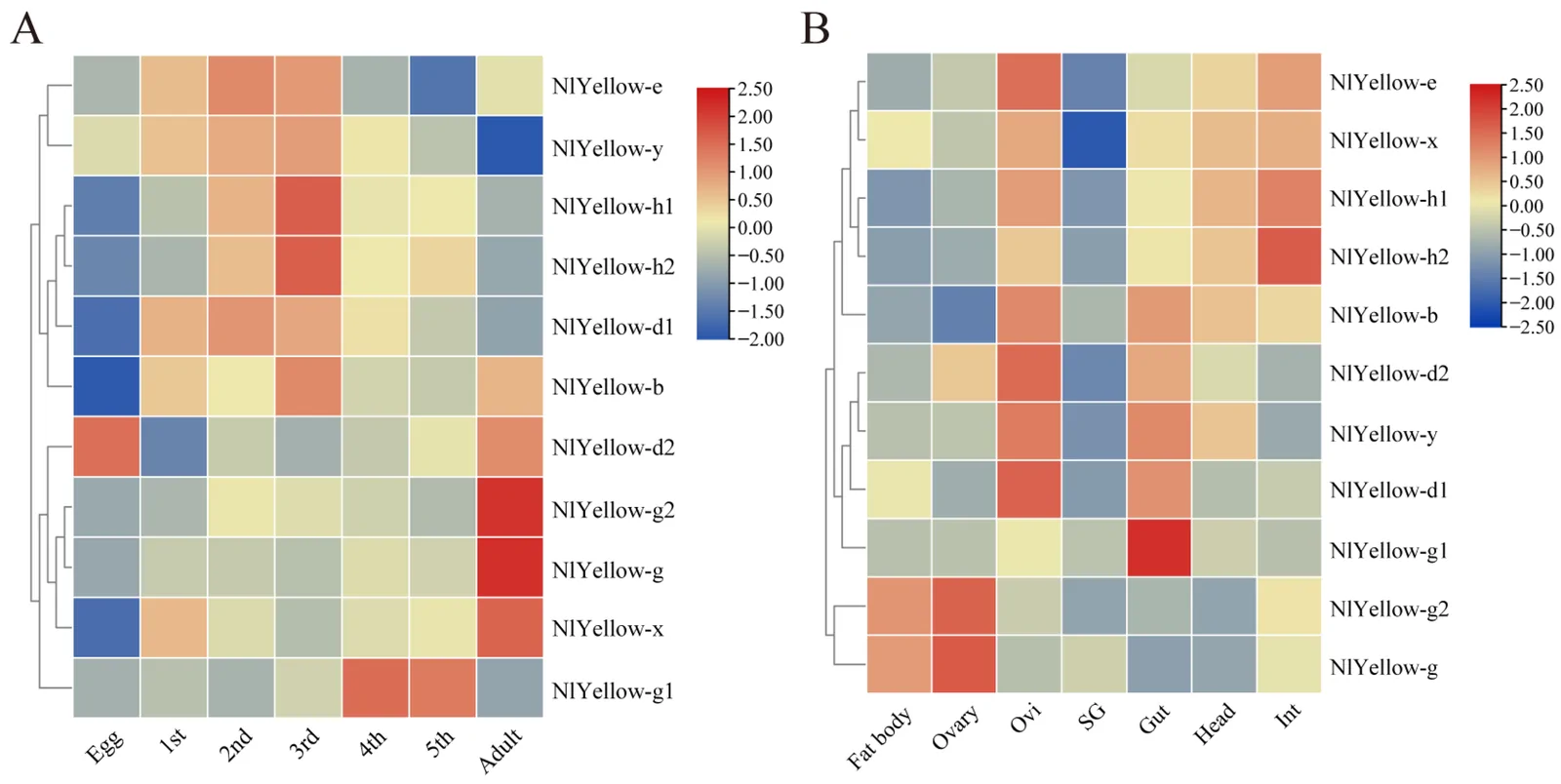
Developmental stage- and tissue-specific expression patterns of *NlYellow* genes. Hierarchically clustered heat maps show the relative expression patterns of *NlYellow* genes at different developmental stages (**A**) and in different tissues (**B**). Heat maps were generated from RPKM values after log2(RPKM + 1) transformation and row-wise normalization. Hierarchical clustering was performed using the Euclidean distance metric and the complete linkage method. Red and blue indicate relatively higher and lower expression levels, respectively, compared with the mean expression level of each gene across samples. Fat body, ovary, ovipositor (Ovi), salivary glands (SG), gut, head, and integument (Int) tissues were dissected from 3-day-old female adults for RNA-seq analysis.

We next examined the expression patterns of the *NlYellow* genes using RNA-seq datasets from different developmental stages of *N. lugens*. Most *NlYellow* genes were broadly expressed at all life stages, suggesting that they may play essential roles in development. Transcript levels of most *NlYellows* were lowest in eggs, although *NlYellow-d2* showed particularly high expression at this stage (Figure 5A). *NlYellow-x*, *-g*, and *-g2* were predominantly expressed in adults, whereas *NlYellow-b*, *-h1*, *-h2*, *-d1*, *-y*, and *-e* showed significantly higher expression at the 2nd or 3rd instar stage (Figure 5A).

RNA-seq data also revealed differences in the tissue-specific expression patterns of the *NlYellow* genes. *NlYellow-g2* and *NlYellow-g* were expressed specifically in the ovary, and *NlYellow-g1* was expressed specifically in the gut. Interestingly, *NlYellow-x*, *NlYellow-h2*, and *NlYellow-h1* were highly expressed in both the ovipositor and the integument (Figure 5B). *NlYellow-e*, *NlYellow-d2*, *NlYellow-y*, *NlYellow-d1*, and *NlYellow-b* were also predominantly expressed in the ovipositor (Figure 5B). *NlYellow-b* showed the highest transcript abundance among all *NlYellow* genes in the available RNA-seq dataset (Table S4), suggesting its potentially important role in *N. lugens* physiology.

**Figure 6:**
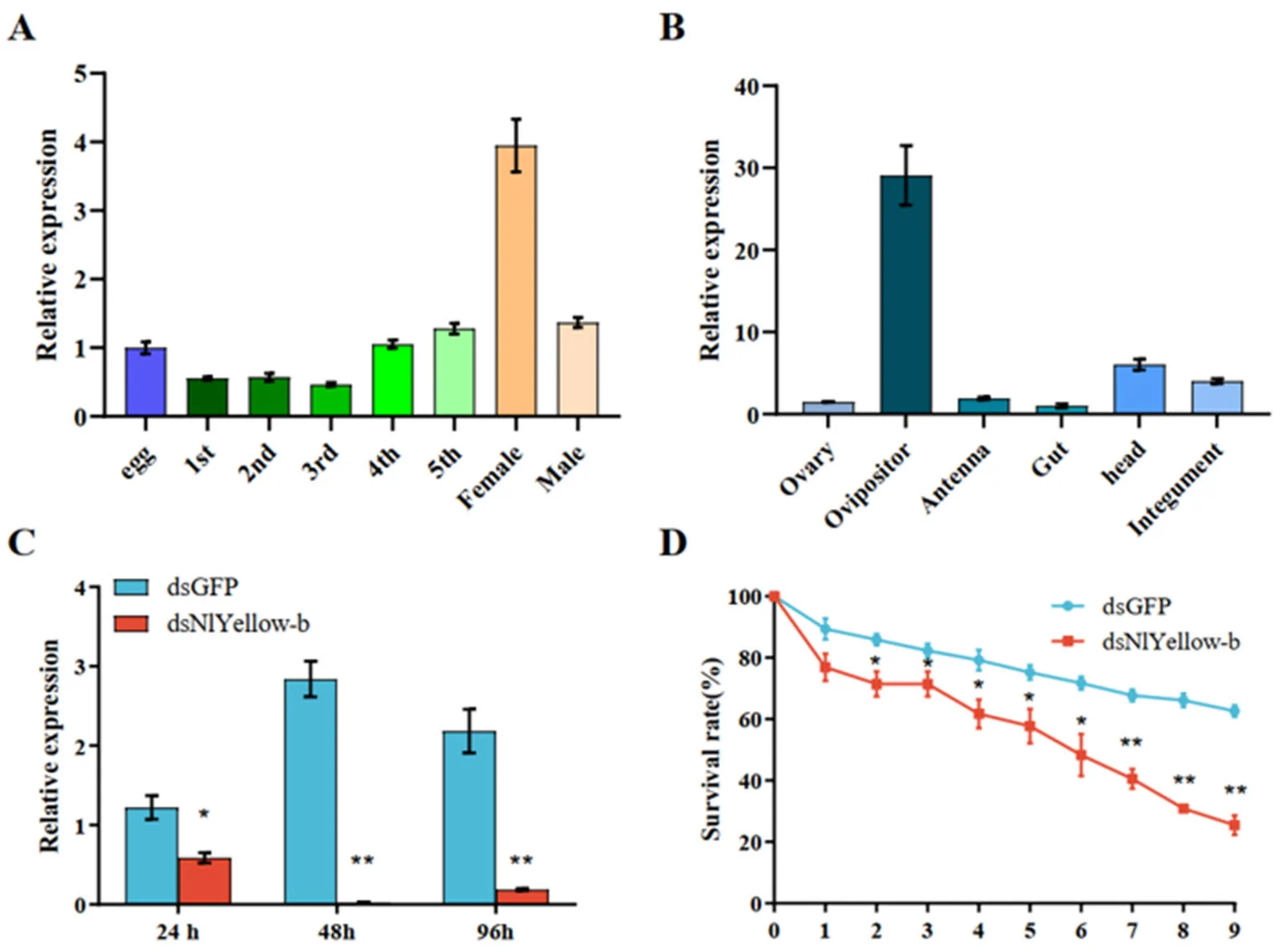
Spatiotemporal expression of *NlYellow-b* and insect survival after *dsNlYellow-b* injection. (**A**) Expression patterns of *NlYellow-b* in eggs, nymphs (1st to 5th instars), female adults, and male adults. (**B**) Expression patterns of *NlYellow-b* in different tissues or body parts. (**C**) Expression of *NlYellow-b* in 4th-instar nymphs at 24, 48, and 96 h post-injection with ds*NlYellow-b* compared with the ds*GFP-*injected controls. (**D**) Survival rate of *N. lugens* post-injection with ds*NlYellow-b* or ds*GFP*. Data are shown as the mean ± SEM from 3 to 5 biological replicates. * *p* < 0.05; ** *p* < 0.01 (Student’s *t*-test).

To confirm the RNA-seq findings, we performed RT–qPCR to precisely quantify *NlYellow-b* expression in *N. lugens*. The results demonstrated striking sexual dimorphism and tissue specificity in *NlYellow-b* expression (Figure 6A). *NlYellow-b* transcript levels were significantly higher in females than in males (Figure 6A) and were highest in the ovipositor among the tissues examined. *NlYellow-b* also exhibited secondary expression peaks in the head and integument (Figure 6B), suggesting that *NlYellow-b* may play pleiotropic roles in multiple physiological processes.

### 3.6. Effects of NlYellow-b Knockdown on Insect Survival

To investigate the function of *NlYellow-b*, we injected fourth-instar nymphs of *N. lugens* with *dsNlYellow-b* or *dsGFP* (as a control). *NlYellow-b* transcript levels were reduced by 51.8%, 99.2%, and 91.4% in *dsNlYellow-b*-injected nymphs compared with the control nymphs at 24, 48, and 96 h, respectively (Figure 6C), confirming the successful silencing of *NlYellow-b* through RNAi. The survival rate was significantly lower in *dsNlYellow-b*-injected nymphs than in the control nymphs from 2 days (71.4% vs. 85.8%) to 9 days (25.4% vs. 62.6%) after injection (Figure 6D), confirming the essential role of *NlYellow-b* in insect survival. 

### 3.7. NlYellow-b Knockdown Disrupts Molting and Wing Development

**Figure 7:**
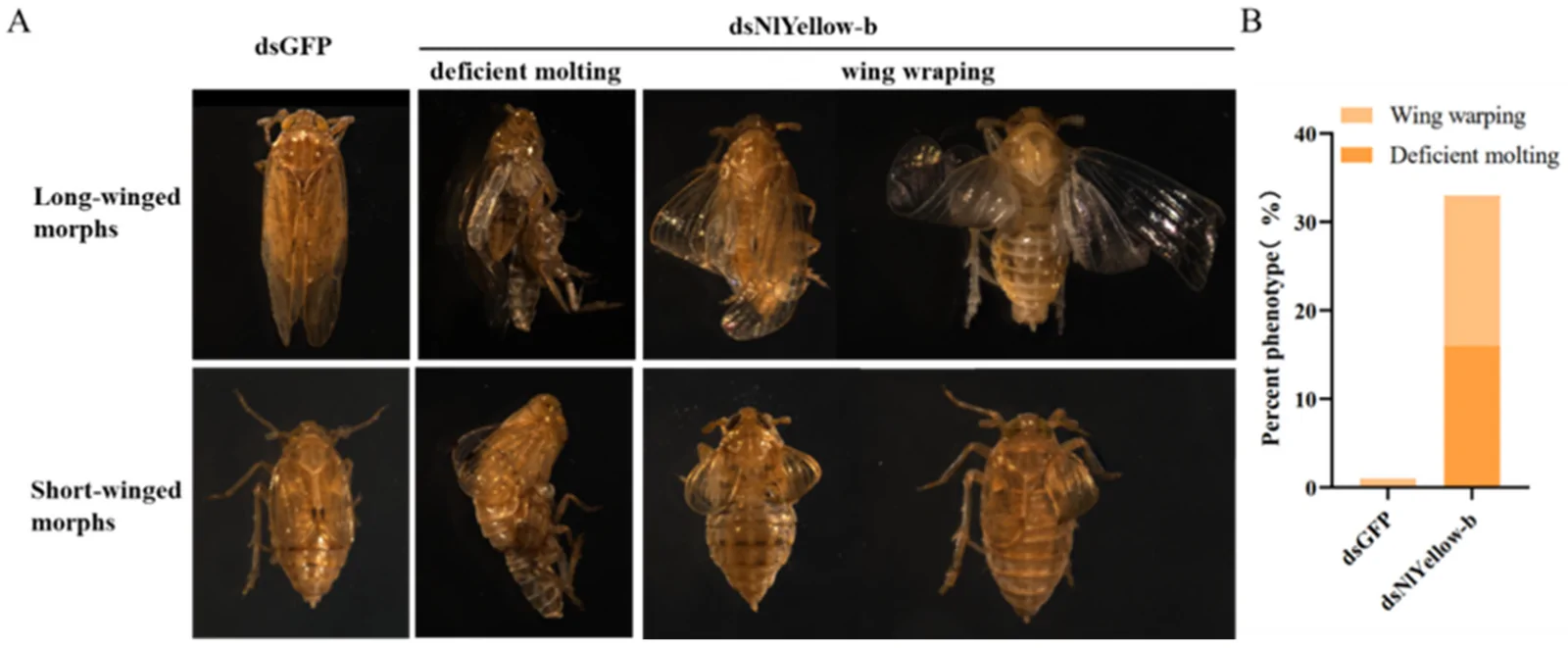
*N. lugens* phenotypes after dsRNA microinjection. Fourth-instar nymphs were injected with dsRNA targeting *NlYellow-b*, with *dsGFP* serving as a non-targeting control. (**A**) The abnormal phenotypes observed after dsRNA injection were categorized into two classes: deficient molting and wing wrapping. Scale bar = 1 mm. (**B**) Percentage of insects that showed mutant phenotypes after dsRNA injection (*n* = 100).

Microinjection of *dsNlYellow-b* resulted in two distinct mutant phenotypes: molting deficiency and wing-wrapping abnormalities. In the molting-deficiency phenotype, the old cuticle remained attached to the abdomen and hind legs, leading to unsuccessful ecdysis during eclosion and subsequent mortality (Figure 7A). This phenotype was observed in 16% of the treated individuals (Figure 7B). Wing-wrapping abnormalities, which affected one or both wings (Figure 7A), occurred at a frequency of 17% (Figure 7B). Both phenotypes were observed in the long-winged and short-winged morphs. These findings demonstrate that *NlYellow-b* knockdown significantly impairs normal development in *N. lugens*.

### 3.8. Effects of NlYellow-b Knockdown on Female Fertility

**Figure 8:**
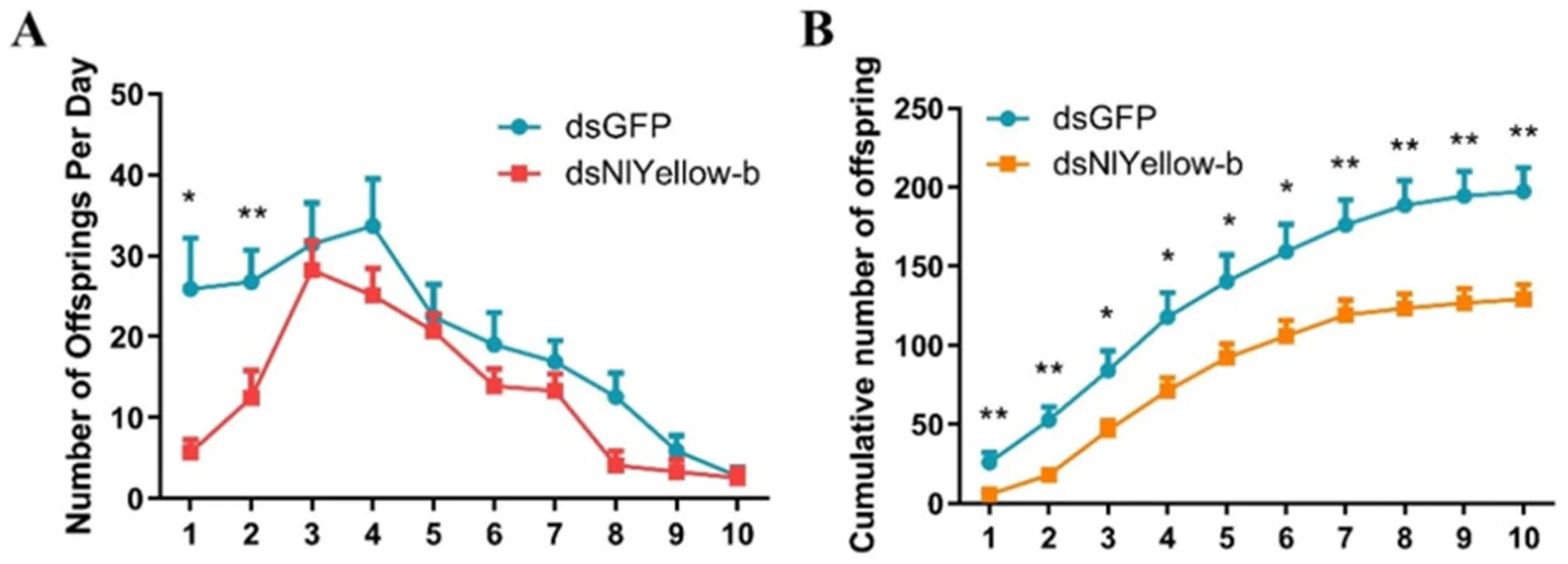
Effects of dsRNA microinjection on offspring production in *N. lugens*. Fifth-instar nymphs were injected with dsRNA targeting *NlYellow-b*, with *dsGFP* serving as a non-targeting control. (**A**) Daily offspring production over 10 days, starting from day 14 after mating. (**B**) Cumulative number of offspring produced from day 14 after mating onward. Asterisks denote statistically significant differences as determined by one-way ANOVA: * *p* < 0.05, ** *p* < 0.01.

Because *NlYellow-b* expression was highest in the ovipositor (Figure 6), we further investigated its role in female reproduction. Cross-mating experiments were performed to evaluate the effect of *NlYellow-b* knockdown on offspring production. Females injected with *dsNlYellow-b* and mated with *dsGFP*-injected males produced significantly fewer offspring than the control group. Over the 10-day observation period, daily offspring production in the *dsNlYellow-b* group was generally lower than that in the *dsGFP* group, with significant differences detected on days 1 and 2 (Figure 8A). Consistently, cumulative offspring production was also significantly reduced in the *dsNlYellow-b* group from the early stage of monitoring onward (Figure 8B). By the end of the observation period, females injected with *dsNlYellow-b* produced an average of 129 nymphs per female, compared with 197 nymphs per female in the *dsGFP* control. These results suggest that *NlYellow-b* is involved in the regulation of female fecundity in *N. lugens*.

## 4. Discussion

Members of the *yellow* gene family show remarkable functional diversity, playing crucial roles in multiple biological processes, including cuticle formation, melanin pigmentation, and mating [9,32,33,34,35,36,37]. Different numbers of *yellow* genes have been identified in a variety of insect species: *Agrotis ipsilon* (7), *B. mori* (7), *D. melanogaster* (14), *Spodoptera frugiperda* (11), *T. castaneum* (14), *Hermetia illucens* (10), and *Apis mellifera* (10) [33,35,38,39,40,41,42]. In the present study, we identified 11 *yellow* genes in *N. lugens*, expanding the known repertoire of these important genes in hemipteran insects. Notably, although the composition of the *yellow* gene family varies among species, all identified Yellow proteins contain a conserved MRJP domain, highlighting the evolutionary conservation of this functionally important domain.

Although several *NlYellow* genes displayed tissue-specific expression patterns, *NlYellow-b* was prioritized for functional analysis because it exhibited the highest transcript abundance among all family members and showed strong enrichment in female reproductive tissues. We acknowledge that additional functional analyses of other *NlYellow* genes, particularly ovary-enriched genes such as *NlYellow-g* and *NlYellow-g2*, may reveal complementary or distinct biological roles. These genes represent promising candidates for future studies.

The *NlYellow* genes of *N*. *lugens* show conserved and lineage-specific features. Phylogenetic analyses have classified the *yellow* genes into nine distinct clades (*yellow-y*, -*b*, -*c*, -*d/e3*, -*e*, -*f*, -*g*, -*g2*, and -*h*), which arose through serial duplication events [27,40]. Notably, *N. lugens* lacks orthologs of *yellow-c* and *yellow-f*, which are present in model insects such as *D. melanogaster* and *T. castaneum* (Figure 1). Comparative genomic studies have shown that seven *yellow* gene clades (*yellow-y*, *-c*, -*d*, -*e*, -*f*, -*g*, and -*h*) were already established prior to the divergence of hemimetabolous and holometabolous insects [28]. Although these genes were present in ancestral lineages, *N. lugens* appears to have undergone secondary loss of *yellow-c* and *yellow-f* during its evolutionary history, potentially owing to functional redundancy or adaptive specialization to its ecological niche. This pattern of gene loss is not unique to *N. lugens*. Similar absences have been observed in diverse insect taxa: the honey bee *A. mellifera* lacks *yellow-c* [39], whereas the black cutworm *A. ipsilon* lacks *yellow-f* [35]. Intriguingly, the black soldier fly *H. illucens* has retained both genes, but mutations in *yellow-c* or *yellow-f* do not have a significant effect on cuticle pigmentation [43]. These findings suggest that *yellow-c* and *yellow-f* may not play major roles in cuticle pigmentation in *H. illucens*, although they may retain redundant, context-dependent, or non-pigmentation-related functions. Different patterns of retention and loss among insect orders provide valuable insights into the functional diversification and evolutionary plasticity of the *yellow* gene family.

RNA-seq analysis showed that *NlYellow-b* was the most highly expressed of the *yellow* genes in *N. lugens* (Table S4), suggesting that it may have a particularly essential biological role. RNAi-mediated knockdown of *NlYellow-b* severely disrupted normal development; pronounced molting defects were observed during the fifth instar (Figure 7), including incomplete ecdysis that led to mortality. Consistent with our findings, CRISPR/Cas9 disruption of *yellow-y* in *S*. *litura* caused partial body deformation in 20.8% of G0 larvae at the fifth instar and abnormal molting in 35.8% at the sixth instar [43]. *T. castaneum* also exhibited molting defects when *yellow-y* was knocked down, with adults failing to shed their pupal cuticle [40]. These results suggest that *yellow* genes have fundamental roles in insect development across taxa but that their specific functions have diversified to meet the unique developmental requirements of different insect species.

Insect wing development is governed by a complex genetic network that involves multiple key regulators, including *Hox* genes, *optix*, and *dopa decarboxylase* (*DDC*) [44,45]. We found that RNAi-mediated knockdown of *NlYellow-b* caused significant wing-wrapping abnormalities that affected one or both wings. Similar defects in wing development were observed in *Blattella germanica*, where *dsBgY-y* impaired forewing and hindwing expansion [46], and in *S. furcifera* upon silencing of *yellow-y* [35]. In addition to its role in wing pigmentation, the *yellow* gene influences aspects of wing morphology, including width, length, and thickness. In *B. anynana*, *yellow* mutants produced extra horizontal laminae on the wing scales [45]. Nonetheless, the molecular mechanisms by which *yellow-y* regulates wing morphology and color patterning remain unresolved and warrant further investigation.

Both RNA-seq and RT–qPCR data indicated that *NlYellow-b* expression was highest in the ovipositor, providing support for the hypothesis that *NlYellow-b* plays a crucial role in female-specific physiological processes, particularly those associated with oviposition. Silencing of *NlYellow-b* in female adults caused a significant reduction in offspring production. This observation is consistent with previous studies highlighting the functional importance of *yellow* genes in insect reproduction. In *Blattella germanica*, knockdown of *BgY-y* not only impaired the response of males to female-emitted contact sex pheromones but also weakened the oviposition ability of RNAi-treated females [46]. Similarly, in *B. mori*, nearly all *yellow-y* mutant embryos failed to hatch without assistance, likely owing to defects in cuticle sclerotization—a process dependent on *yellow-y* function [47]. Consistent with this notion, studies on other insects have demonstrated critical roles of *yellow* genes in eggshell formation and protection. In *T. castaneum*, both *TcY-g* and *TcY-g2* are essential for the maintenance of chorion rigidity and integrity, which are crucial for resistance to mechanical stress and rehydration during ovulation and egg activation [48]. Likewise, in *Aedes albopictus*, *AalY-g* and *AalY-g2* regulate the timing of eggshell darkening and are required for exochorion integrity, as well as the proper rigidity, the correct morphology, and formation of the outer endochorion—a structure vital for egg desiccation resistance. *AalY-y* also participates in the tyrosine-mediated melanin biosynthesis pathway, contributing to chorion melanization and ensuring correct morphology of the outer endochorion, which is presumably necessary for egg desiccation resistance [49]. In summary, our results reveal that *NlYellow-b* acts as a key regulator of female reproduction in *N. lugens*. The marked enrichment of *NlYellow-b* transcripts in the ovipositor and the reduction in offspring production following RNAi suggest that this gene contributes to female reproductive performance. However, the precise anatomical or physiological basis of this phenotype remains unclear. Future studies combining histological observations and ultrastructural analyses of the ovipositor and reproductive tissues will be required to elucidate the underlying mechanisms.

These findings add to the growing evidence that *yellow* genes have both lineage-specific and functionally conserved roles in insect reproduction, making them potential targets for pest control strategies. Overall, this work advances our understanding of a key gene family in a major rice pest and highlights *NlYellow-b* as a promising molecular target for RNAi-based control strategies, thereby contributing to the broader effort to develop sustainable, species-specific pest management solutions.

## Figures and Tables

**Table 1:** Structural and biochemical characteristics of yellow proteins in *Nilaparvata lugens*. MW, Molecular weight; PI, Theoretical isoelectric point; II, Instability index; AI, Aliphatic index; GRAVY, Grand average of hydropathicity.

Gene ID	Locus	Chromosome Location	Strand	Genomic (bp)	CDS (bp)	Amino Acids (aa)	MW (kDa)	PI	II	AI	GRAVY	Exon_ Num	Intron_ Num
*NlYellow-y*	LOC111043567	Ch1:49229290-49230309	reverse	1020	945	314	35.96	5.23	33.29	72.36	−0.483	1	0
*NlYellow-g1*	LOC111064326	Ch8:1862228-1899585	forward	37,358	2232	743	83.60	5.03	41.34	88.88	−0.306	12	11
*NlYellow-e*	LOC111062511	Ch8:14285129-14362049	reverse	76,921	1266	421	46.99	6.19	47.7	84.11	−0.232	5	4
*NlYellow-g2*	LOC111062260	Ch8: 14315871-14396332	forward	80,462	1146	381	42.38	8.21	27.74	92.41	0.042	6	5
*NlYellow-g*	LOC111062320	Ch8: 14371471-14381246	reverse	9776	1173	390	43.63	9.08	33.61	90.49	−0.039	4	3
*NlYellow-d1*	LOC111062051	Ch8: 18074907-18090745	forward	15,839	870	289	32.85	6.43	31.06	87.38	−0.193	6	5
*NlYellow-x*	LOC111063562	Ch8: 18481060-18487793	reverse	6734	1302	433	48.55	6.19	52.23	93.37	−0.1	1	0
*NlYellow-d2*	LOC111061594	Ch8: 40243499-40284501	reverse	41,003	1341	446	50.50	7.61	38.46	76.48	−0.232	6	5
*NlYellow-h2*	LOC111060168	Ch8: 46964543-46972313	forward	7771	1560	519	58.57	8	44.9	76.74	−0.429	3	2
*NlYellow-h1*	LOC120352860	Ch8: 47098331-47112244	reverse	13,914	1302	433	49.25	8.02	39.45	78.27	−0.442	4	3
*NlYellow-b*	LOC111061161	ChX: 13817413-13828097	reverse	10,685	1251	416	47.02	5.44	26.27	83.65	−0.365	2	1

## Data Availability

The data presented in this study will be made available on request.

## Supplementary Materials

The following supporting information can be downloaded at https://www.mdpi.com/article/10.3390/insects17080766/s1. Table S1: Amino acid sequences of the NlYellows, BmYellows, TcYellow, sDmYellows and Aayellows. Table S2: Primers used for the PCR and qRT-PCR analysis. Table S3: The ten predicted motif categories detected using MEME for the 11 NlYellows in *N. lugens*. Table S4: Expression levels of *Nlyellow* genes determined by RNA-seq.
